# The giant increase of stiffness of inhomogeneous rods and beams

**DOI:** 10.1038/s41598-024-69396-2

**Published:** 2025-05-30

**Authors:** A. A. Kolpakov, A. G. Kolpakov

**Affiliations:** 1 University of Neuchatel, Rue Emile-Argand 11, 2000 Neuchâtel, Switzerland; 2 SysAn (System Analysis in Engineering), Ul. Aleksandra Nevskogo, 12a, Novosibirsk, Russia 630075

**Keywords:** Materials science, Computational methods, Applied mathematics

## Abstract

We demonstrate that stiffnesses of an inhomogeneous beam of coaxial structure coincide with the ones predicted by classical Bernoulli–Euler and Saint-Venant theories if and only if the indicated below conditions on the local Poisson’s ratio are satisfied. If the conditions are not satisfied, the stiffnesses of the inhomogeneous beam exceed the stiffnesses predicted by classical theories. The difference in Poisson’s ratios of the components of the rod/beam can result in a giant increase in stiffness when using materials possessing a negative Poisson’s ratio.

## Introduction

The classical Bernoulli–Euler and Saint-Venant beam theories were presented in^1–3^, see also^4^. While the local deformations of the beam are very different in the classical theories (Bernoulli–Euler theory ignores and the Saint-Venant theory accounts the transverse deformations of the beam), the stiffnesses predicted by both the theories turn out to be the same. Following the classical theories, advanced rod/beam theories were developed, see^5–8^ and references herein. At the last time, the inhomogeneous beams (in particular, laminated beams, which are a special case of the coaxial beams) attract attention of the researchers, see, e.g.,^9,10^. The general theory of inhomogeneous beams was developed by using the ideas of the asymptotic homogenization theory^11^, see, e.g.,^12–14^ and references herein. However, the relationship of the stiffnesses calculated in various ways is not fully clarified until now.

**Figure 1:**
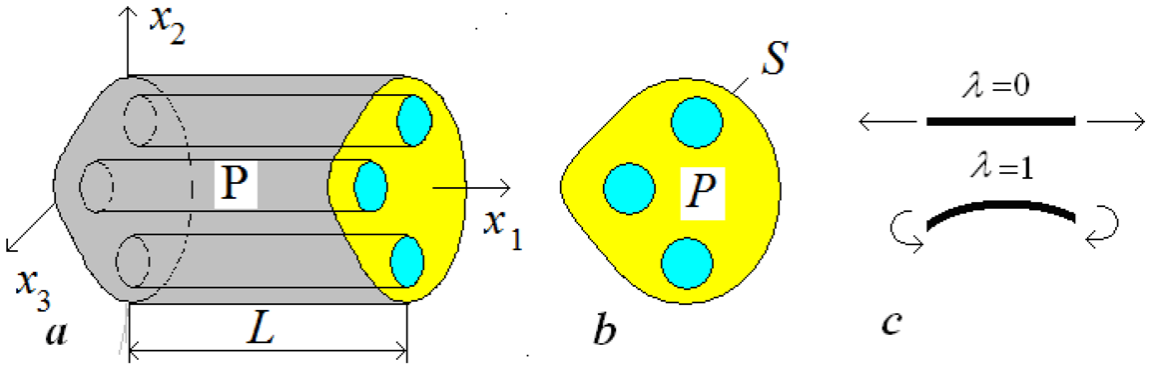
The periodicity cell ${\mathbf{P}}$ of the cylindrical rod/beam—(**a**); cross section $P$—(**b**); deformations of the rod ($\lambda = 0$) and beam ($\lambda = 1$)—(**c**).

We consider a beam which axis coincides with the $Ox_{1}$-axis of the standard orthonormal coordinate system $Ox_{1} x_{2} x_{3}$ (${\mathbf{e}}_{1} ,{\mathbf{e}}_{2} ,{\mathbf{e}}_{3}$ are the unit basis vectors). In accordance with^12–14^, the displacement of the beam ${\mathbf{u}} \approx u_{1} (x_{1} ){\mathbf{e}}_{1} + w_{A} (x_{1} ){\mathbf{e}}_{A} + \varepsilon {\mathbf{N}}^{0} ({\mathbf{x}}/\varepsilon )u_{1}^{\prime } (x_{1} ) + \varepsilon {\mathbf{N}}^{1A} ({\mathbf{x}}/\varepsilon )w_{A}^{\prime \prime } (x_{1} )$, where $u_{1} (x_{1} )$ is the overall axial displacement and $w_{A} (x_{1} )$ ($A = 2,3$) are the overall normal deflections of the beam. The functions ${\mathbf{N}}^{0}$ and ${\mathbf{N}}^{1A}$ are solutions to the so-called periodicity cell problem^12^:

1 $$\left\{ \begin{gathered} (a_{ijkl} (y_{2} ,y_{3} )N_{k,l}^{\lambda A} + ( - 1)^{\lambda } a_{ij11} (y_{2} ,y_{3} )y_{A}^{\lambda } )_{,jy} = 0{\text{ in }}{\mathbf{P}}, \hfill \\ (a_{ijkl} (y_{2} ,y_{3} )N_{k,l}^{\lambda A} + ( - 1)^{\lambda } a_{ij11} (y_{2} ,y_{3} )y_{A}^{\lambda } )n_{\alpha } = 0{\text{ on }}\Gamma , \hfill \\ {\mathbf{N}}^{\lambda A} ({\mathbf{y}}){\text{ periodic in }}y_{1} \hfill \\ \end{gathered} \right.$$

which describes the local deformations of the periodicity cell ${\mathbf{P}}$ of the beam ($\Gamma$ is the lateral surface of ${\mathbf{P}}$). Problem (1) with $\lambda = 0$ corresponding to the overall axial deformations, with $\lambda = 1$—the overall bending in the $Oy_{1} y_{A}$-plane, see Fig. 1c. Following^11^, we use the original ${\mathbf{x}}$ and the local ${\mathbf{y}} = {\mathbf{x}}/\varepsilon$ coordinates.

Stiffnesses $d_{AB}^{\lambda + \mu }$ of the beam ($\lambda ,\mu = 0,1$, $A,B = 2,3$) are calculated according to the formula^12–14^

2 $$d_{AB}^{\lambda + \mu } = ( - 1)^{\mu } L^{ - 1} \int\limits_{{\mathbf{P}}} {(a_{11kl} ({\mathbf{y}})N_{k,ly}^{\lambda A} + ( - 1)^{\lambda } a_{1111} ({\mathbf{y}})y_{A}^{\lambda } )y_{B}^{\mu } d{\mathbf{y}}.}$$

Formula (2) determines the tensile stiffness $d_{{}}^{0}$ for $\lambda = \mu = 0$, (it does not have the index $AB$) and bending stiffnesses for $\lambda = \mu = 1$.

We will analyze problem (1) for a beam of coaxial structure made of isotropic material(s) and demonstrate that the stiffnesses (2) coincide with the classical ones if and only if the local Poisson’s ratio satisfies a condition derived below. If the condition is not satisfied, the stiffnesses (2) are greater than the stiffnesses of the corresponding homogeneous beam. Our numerical calculations show that the difference of the stiffnesses can be very large.

## Rods/beams of coaxial structure. Transition to two-dimensional problems

The beam under consideration is a thin inhomogeneous cylinder of characteristic diameter $\varepsilon$ made of isotropic material, Fig. 1a,b. Its elastic constants dependent on $y_{2} ,y_{3}$ only (do not depend on $y_{1}$): $a_{ijkl} = a_{ijkl} (y_{2} ,y_{3} )$ and the periodicity cell (representative fragment) of the beam can be chosen as ${\mathbf{P}} = [0,L] \times P$, Fig. 1a, where $L$ is an arbitrary positive number, $P$ is the cross section of the rod/beam, Fig. 1b.

For the beam under consideration, solution to problem (1) has the form ${\mathbf{N}}^{\lambda A} ({\mathbf{y}}) = {\mathbf{N}}^{\lambda A} (y_{2} ,y_{3} )$. Since the material is isotropic, we obtain from the first ($i = 1$) equation in (1) that $N_{1}^{\lambda A} (y_{2} ,y_{3} ) = 0$. For $i = 2,3$, we have from (1) the following planar problem ($\alpha ,\beta ,\gamma ,\delta = 2,3$):

3 $$\left\{ \begin{gathered} (a_{\alpha \beta \gamma \delta } (y_{2} ,y_{3} )N_{\gamma ,\delta }^{\lambda A} + ( - 1)^{\lambda } a_{\alpha \beta 11} (y_{2} ,y_{3} )y_{A}^{\lambda } )_{,\beta y} = 0{\text{ in }}P, \hfill \\ (a_{\alpha \beta \gamma \delta } (y_{2} ,y_{3} )N_{\gamma ,\delta }^{\lambda A} + ( - 1)^{\lambda } a_{\alpha \beta 11} (y_{2} ,y_{3} )y_{A}^{\lambda } )n_{\beta } = 0{\text{ on }}S. \hfill \\ \end{gathered} \right.$$

The equalities in (3) are satisfied if the expression in brackets in (3) is equal to zero. Let us ask ourselves: are there any strains $e_{\gamma \delta }^{A}$ (possibly inconsistent) that deliver a solution to the following algebraic system of equations?

4 $$a_{\alpha \beta \gamma \delta } (y_{2} ,y_{3} )e_{\gamma \delta }^{A} + ( - 1)^{\lambda } a_{\alpha \beta 11} (y_{2} ,y_{3} )y_{A}^{\lambda } = 0.$$

Equation (4) in coordinate form is the following:

5 $$a_{2222} e_{22}^{A} + a_{2233} e_{33}^{A} = - ( - 1)^{\lambda } a_{2211} (y_{2} ,y_{3} )y_{A}^{\lambda } , \, a_{3322} e_{22}^{A} + a_{3333} e_{33}^{A} = - ( - 1)^{\lambda } a_{3311} (y_{2} ,y_{3} )y_{A}^{\lambda } , \, a_{2323} e_{23}^{A} = 0.$$

For isotropic materials, elastic constants

6 $$a_{2222} = \frac{E(1 - \nu )}{{(1 + \nu )(1 - 2\nu )}}, \, a_{1122} = \frac{E\nu }{{(1 + \nu )(1 - 2\nu )}},\;a_{2222} + a_{2233} = \frac{E}{(1 + \nu )(1 - 2\nu )},$$

where $E = E(y_{2} ,y_{3} )$ is the Young’s modulus and $\nu = \nu (y_{2} ,y_{3} )$ is the Poisson’s ratio^4^. Solution to (5) with the coefficients (6) is the following ($\delta_{\alpha \beta }^{{}}$ is Knonecker’s delta):

7 $$e_{\alpha \beta }^{\lambda A} = - ( - 1)^{\lambda } \delta_{\alpha \beta } \nu (y_{2} ,y_{3} )y_{A}^{\lambda } \;\left( {\lambda ,\mu = 0,1} \right).$$

Solution (7) for homogeneous beams has been known since the time of Saint-Venant^3^. Also, solution (7) remains valid for inhomogeneous beams.

Taking into account (4) and (7), problem (3) can be rewritten in the form

8 $$\left\{ \begin{gathered} (a_{\alpha \beta \gamma \delta } (y_{2} ,y_{3} )(N_{\gamma ,\delta }^{\lambda A} - e_{\gamma \delta }^{\lambda A} ))_{,\beta } = 0{\text{ in }}P, \\ a_{\alpha \beta \gamma \delta } (y_{2} ,y_{3} )(N_{\gamma ,\delta }^{\lambda A} - e_{\gamma \delta }^{\lambda A} )n_{\beta } = 0{\text{ on }}S. \\ \end{gathered} \right.$$

For the cylindrical periodicity (representative) cell $L^{ - 1} \int\limits_{{\mathbf{P}}} {d{\mathbf{y}}} = \int\limits_{P} {dy_{2} dy_{3} }$, and thus the general formula (2) takes the form

9 $$d_{AB}^{\lambda + \mu } = ( - 1)^{\mu } \int\limits_{P} {(a_{11\alpha \beta } ({\mathbf{y}})N_{\alpha ,\beta }^{\lambda A} + ( - 1)^{\lambda } a_{1111} ({\mathbf{y}})y_{A}^{\lambda } )y_{B}^{\mu } dy_{2} dy_{3} .}$$

If the strains (7) are compatible, i.e., there exists displacement ${\mathbf{N}}^{\lambda A} ({\mathbf{y}})$, such that

10 $$N_{\alpha ,\beta }^{\lambda A} = - ( - 1)^{\lambda } \delta_{\alpha \beta } \nu (y_{2} ,y_{3} )y_{A}^{\lambda } ,$$

then this ${\mathbf{N}}^{\lambda A} ({\mathbf{y}})$ is the solution to problem (3). Substituting (10) into (9) leads to the equality

$$d_{AB}^{\lambda + \mu } = ( - 1)^{\lambda + \mu } \int\limits_{P} {(a_{1111} (y_{2} ,y_{3} ) - a_{1122} (y_{2} ,y_{3} )\nu(y_{2} ,y_{3} ) - a_{1133} (y_{2} ,y_{3} )\nu (y_{2} ,y_{3} ))y_{2}^{\lambda + \mu } dy_{2} dy_{3} } .$$

From (6) it follows that $a_{1111} - a_{1122} \nu - a_{1133} \nu = E$, then the equation above takes the form (hereafter “$\triangleq$ ” means “equal by definition”)

11 $$d_{AB}^{\lambda + \mu } = D_{AB}^{\lambda + \mu } \triangleq ( - 1)^{\lambda + \mu } \int\limits_{P} {E(y_{2} ,y_{3} )y_{A}^{\lambda } y_{B}^{\mu } dy_{2} dy_{3} .}$$

Hereafter, $D_{AB}^{\nu + \mu }$ mean the classical stiffnesses, in particular, $D^{0} = \int\limits_{P} {E(y_{2} ,y_{3} )dy_{2} dy_{3} }$ is the tensile stiffness, $D_{AA}^{2} = \int\limits_{P} {E(y_{2} ,y_{3} )y_{A}^{2} dy_{A} dy_{3} }$ ($A = 2,3$) are the bending stiffnesses.

Equality (11) means that if the strains (7) are compatible, the stiffnesses in the classical and the asymptotic theories coincide. So, it can be concluded that the compatibility of strains $e_{\alpha \beta }^{\lambda A}$ (7) is a key point in the analysis of rod/beam stiffnesses.

## The case of compatible strains (7)

Let’s denote $Inc \triangleq \frac{{\partial^{2} }}{{\partial y_{3}^{2} }} + \frac{{\partial^{2} }}{{\partial y_{22}^{2} }} - 2\frac{{\partial^{2} }}{{\partial y_{2} \partial y_{3} }}$ the operator characterizing the incompatibility of planar strains^4^. The strains are compatible if and only if $Inc(e_{\alpha \beta } ) = 0$^4^. For the strains $e_{\alpha \beta }^{\lambda A}$ $= - ( - 1)^{\lambda } \delta_{\alpha \beta } \nu (y_{2} ,y_{3} )y_{A}^{\lambda }$ (7), the equality $Inc(e_{\alpha \beta } ) = 0$ takes the form

12 $$\Delta (\nu (y_{2} ,y_{3} )y_{A}^{\lambda } ) = 0\;\left( {\lambda = 0,1} \right),$$

Equality (12) is a sufficient condition for the stiffness of the considered composite beam to coincide with the classical ones. Below, we will show that condition (12) is also necessary for the stiffness of the rod/beam to coincide with the classical ones.

## The case of incompatible strains (7). Non-classical stiffnesses

Let us consider the problem (8), (9) for incompatible $e_{\alpha \beta }^{\lambda A}$ (7). If (12) fails, then $N_{\alpha ,\beta }^{\lambda A} \ne e_{\alpha \beta }^{\lambda A}$ for any displacements ${\mathbf{N}}^{\lambda A} (y_{2} ,y_{3} )$. Let’s write the problem under consideration with respect to the function $N_{\alpha ,\beta y}^{\lambda A} - e_{\alpha \beta }^{\lambda A}$.

We write (9) as follows:

$$d_{AB}^{\lambda + \mu } = ( - 1)^{\mu } \int\limits_{P} {(a_{11\alpha \beta } (y_{2} ,y_{3} )(N_{\alpha ,\beta y}^{\lambda A} - e_{\alpha \beta }^{\lambda A} ) + a_{11\alpha \beta } (y_{2} ,y_{3} )e_{\alpha \beta }^{\lambda A} + ( - 1)^{\lambda } a_{1111} (y_{2} ,y_{3} )y_{A}^{\lambda } )y_{B}^{\mu } dy_{2} dy_{3} .}$$

The integral of the last two terms is equal to the classical stiffness $D_{AB}^{\lambda + \mu }$ (11). Then

13 $$d_{AB}^{\lambda + \mu } = D_{AB}^{\lambda + \mu } + ( - 1)^{\mu } \int\limits_{P} {a_{11\alpha \beta } (y_{2} ,y_{3} )(N_{\alpha ,\beta }^{\lambda A} - e_{\alpha \beta }^{\lambda A} )y_{B}^{\mu } dy_{2} dy_{3} .}$$

The problem (8) is yet written in the term of the function $N_{\alpha ,\beta y}^{\lambda A} - e_{\alpha \beta }^{\lambda A}$.

Stresses $\sigma_{\alpha \beta }$ corresponding to the problem (8) are the following:

14 $$\sigma_{\alpha \beta } = a_{\alpha \beta \gamma \delta } (y_{2} ,y_{3} )(N_{\gamma ,\delta }^{\lambda A} - e_{\gamma \delta }^{\lambda A} ) = \sigma_{\alpha \beta }^{0} + \sigma_{\alpha \beta }^{i} ,$$

where

15 $$\sigma_{\alpha \beta }^{0} = a_{\alpha \beta \gamma \delta } (y_{2} ,y_{3} )N_{\gamma ,\delta } , \, \sigma_{\alpha \beta }^{i} = - a_{\alpha \beta \gamma \delta } (y_{2} ,y_{3} )e_{\gamma \delta } .$$

Let us introduce a function $\Phi (y_{2}^{{}} ,y_{3}^{{}} )$ ($\Phi = \Phi_{{}}^{\lambda A} )$ (Airy-type function) by the equalities

16 $$\sigma_{22} = \frac{{\partial^{2} \Phi }}{{\partial y_{3}^{2} }}, \, \sigma_{23} = - \frac{{\partial^{2} \Phi }}{{\partial y_{2} \partial y_{3} }}, \, \sigma_{33} = \frac{{\partial^{2} \Phi }}{{\partial y_{2}^{2} }}.$$

The quantities $\sigma_{\alpha \beta }$ (16) satisfy the equilibrium equations $\sigma_{\alpha \beta ,\beta } = 0$ corresponding to (8).

The compatibility condition may be written in the terms of stresses. For isotropic material it has the form^4^

17 $$Inc(\sigma_{\alpha \beta } ) \triangleq \frac{{\partial^{2} }}{{\partial y_{3}^{2} }}\left[ {\frac{1 + \nu }{E}(\sigma_{22} - v(\sigma_{22} + \sigma_{33} ))} \right] + \frac{{\partial^{2} }}{{\partial y_{22}^{2} }}\left[ {\frac{1 + \nu }{E}(\sigma_{33} - v(\sigma_{22} + \sigma_{33} ))} \right] - 2\frac{{\partial^{2} }}{{\partial y_{2} \partial y_{3} }}\left[ {\frac{1 + \nu }{E}\sigma_{23} } \right] = 0.$$

Substituting into (17) $\sigma_{22}^{{}} , \, \sigma_{23}^{{}} , \, \sigma_{33}^{{}}$ in accordance with (16), we obtain

18 $$Inc(\sigma_{\alpha \beta } ) = \frac{{\partial^{2} }}{{\partial y_{3}^{2} }}\left[ {\frac{1 + \nu }{E}\left( {\frac{{\partial^{2} \Phi }}{{\partial y_{3}^{2} }} - v\Delta \Phi } \right)} \right] + \frac{{\partial^{2} }}{{\partial y_{2}^{2} }}\left[ {\frac{1 + \nu }{E}\left( {\frac{{\partial^{2} \Phi }}{{\partial y_{2}^{2} }} - v\Delta \Phi } \right)} \right] + 2\frac{{\partial^{2} }}{{\partial y_{2} \partial y_{3} }}\left[ {\frac{1 + \nu }{E}\frac{{\partial^{2} \Phi }}{{\partial y_{2} \partial y_{3} }}} \right] \triangleq L\Phi .$$

By virtue of (14), $Inc(\sigma_{\alpha \beta } )$ = $Inc(\sigma_{\alpha \beta }^{0} + \sigma_{\alpha \beta }^{i} )$. Since operator $Inc$ is linear and stresses $\sigma_{\alpha \beta }^{0}$ are compatible, then $Inc(\sigma_{\alpha \beta } )$ = $Inc(\sigma_{\alpha \beta }^{i} )$. Let's calculate $Inc(\sigma_{\alpha \beta }^{i} )$ (17) for $\sigma_{\gamma \delta }^{i}$ (15). Substituting $e_{\gamma \delta }^{{}} = e_{\alpha \beta }^{\lambda A} = - ( - 1)^{\lambda } \delta_{\alpha \beta } \nu (y_{2} ,y_{3} )y_{A}^{\lambda }$ (7) into (15), we get $\sigma_{\gamma \delta }^{i} = a_{\gamma \delta \alpha \alpha } (y_{2} ,y_{3} )( - 1)^{\lambda } \nu (y_{2} ,y_{3} )y_{A}^{\lambda }$. From these equations, we have

19 $$\begin{gathered} \sigma_{22}^{i} = (a_{2222} (y_{2} ,y_{3} ) + a_{2233} (y_{2} ,y_{3} ))( - 1)^{\lambda } \nu (y_{2} ,y_{3} )y_{A}^{\lambda } , \hfill \\ \sigma_{33}^{i} = (a_{3322} (y_{2} ,y_{3} ) + a_{3333} (y_{2} ,y_{3} ))( - 1)^{\lambda } \nu (y_{2} ,y_{3} )y_{A}^{\lambda } {. } \hfill \\ \end{gathered}$$

For isotropic material $a_{2222} + a_{2233} = a_{3333} + a_{3322} = \frac{E}{(1 + \nu )(1 - 2\nu )}$ and we obtain from (19) that $\sigma_{22}^{i} = \sigma_{33}^{i}$ $= ( - 1)^{\lambda } \frac{E\nu }{{(1 + \nu )(1 - 2\nu )}}y_{A}^{\lambda }$. Also, $\sigma_{23}^{i} = \sigma_{32}^{i} = 0.$ Substituting these $\sigma_{\alpha \beta }^{i}$ into (17), we have $Inc(\sigma_{\alpha \beta }^{i} ) = ( - 1)^{\lambda } \Delta [\nu y_{A}^{\lambda } ]$. Finally, we obtain from (18) that $L\Phi^{\lambda A} = ( - 1)^{\lambda } \Delta [\nu y_{A}^{\lambda } ]$.

Let’s write the boundary condition $\sigma_{\alpha \beta } n_{\beta } = 0$ on $S$ (8) in the term of function $\Phi$. Substituting $\sigma_{22}$, $\sigma_{23}$, $\sigma_{33}$ according to (16), we obtain $\frac{{\partial^{2} \Phi }}{{\partial y_{3}^{2} }}n_{2} - \frac{{\partial^{2} \Phi }}{{\partial y_{2} \partial y_{3} }}n_{3} = 0$ and $- \frac{{\partial^{2} \Phi }}{{\partial y_{2} \partial y_{3} }}n_{2} + \frac{{\partial^{2} \Phi }}{{\partial y_{2}^{2} }}n_{3} = 0$ on $S$. Since $\frac{\partial }{{\partial y_{3} }}n_{2} - \frac{\partial }{{\partial y_{2} }}n_{3} = \frac{d}{ds}$ is the operator of differentiation along the boundary $S$, we obtain $\frac{d}{ds}\frac{\partial \Phi }{{\partial y_{3} }} = 0$, $\frac{d}{ds}\frac{{\partial^{2} \Phi }}{{\partial y_{2} }} = 0$ on $S$. From here we obtain after some computations $\, \frac{\partial \Phi }{{\partial {\mathbf{n}}}} = \Phi = 0$ on $S$.

### Non-classical corrections to the stiffnesses

Equality (13) means that $d_{AB}^{\lambda + \mu } = D_{AB}^{\lambda + \mu } + \delta D_{AB}^{\lambda + \mu }$, where $D_{AB}^{\lambda + \mu }$ are the classical stiffnesses (11), and $\delta D_{AB}^{\lambda + \mu } = \int\limits_{P} {a_{11\alpha \beta } (y_{2} ,y_{3} )(N_{\alpha ,\beta }^{\lambda A} - e_{\alpha \beta }^{\lambda 2} )y_{B}^{\mu } dy_{2} dy_{3} }$ are the (non-classical) corrections. Let’s investigate them further. With regard to (6), we have

20 $$\delta D_{AB}^{\lambda + \mu } = ( - 1)^{\mu } \int\limits_{S} {\frac{E\nu }{{(1 + \nu )(1 - 2\nu )}}\left[ {(N_{2,2}^{\lambda A} - e_{22}^{\lambda A} ) + (N_{3,3}^{\lambda A} - e_{33}^{\lambda A} )} \right]y_{B}^{\mu } dy_{2} dy_{3} .}$$

Using (17), we express $N_{2,2}^{\lambda A} - e_{22}^{\lambda A}$ and $N_{3,3}^{\lambda A} - e_{33}^{\lambda A}$ from (14) as follows:

$$N_{2,2}^{\lambda A} - e_{22}^{\lambda A} = \frac{{[\sigma_{22} (1 - \nu ) - \sigma_{33} \nu](1 + \nu )}}{E},\;N_{3,3}^{\lambda A} - e_{33}^{\lambda A} = \frac{{[(1 - \nu )\sigma_{33} - \sigma_{22} \nu](1 + \nu )}}{E}.$$

Substituting here $\sigma_{22}$ and $\sigma_{33}$ according to (16), we arrive at the equalities **(**$\Phi = \Phi^{\lambda A}$**)**

21 $$N_{2,2}^{\lambda A} - e_{22}^{\lambda A} = \frac{{\left[ {\frac{{\partial^{2} \Phi }}{{\partial y_{3}^{2} }}(1 - \nu ) - \frac{{\partial^{2} \Phi }}{{\partial y_{22}^{2} }}\nu} \right](1 + \nu )}}{E}, \, N_{3,3}^{\lambda A} - e_{33}^{\lambda A} = \frac{{\left[ {(1 - \nu )\frac{{\partial^{2} \Phi }}{{\partial y_{22}^{2} }} - \frac{{\partial^{2} \Phi }}{{\partial y_{3}^{2} }}\nu} \right](1 + \nu )}}{E}.$$

Substituting (21) into (20), we find that the non-classical correction to the stiffness $D_{AB}^{\nu + \mu }$ is

22 $$\delta B_{AB}^{\lambda + \mu } = ( - 1)^{\mu } \int\limits_{P} {\nu (y_{2} ,y_{3} )\Delta \Phi^{\lambda A} y_{B}^{\mu } dy_{2} dy_{3} } .$$

It is seen that the non-classical correction takes place both for the tensile stiffness $D_{{}}^{0}$ (the case $\lambda = \mu = 0$) and for bending stiffnesses $D_{22}^{2}$, $D_{33}^{2}$ (the case $\lambda = \mu = 1$).

### The problem of non-classical corrections to stiffness

Finally, we arrive at the following boundary-value problem **(**$\Phi = \Phi^{\lambda A}$, $L\Phi$ is defined in (18)):

23 $$\left\{ \begin{gathered} L\Phi = ( - 1)^{\lambda } \Delta [\nu(y_2,y_3) y_{A}^{\lambda } ]{\text{ in }}D, \hfill \\ \Phi = \frac{\partial \Phi }{{\partial {\mathbf{n}}}} = 0{\text{ on }}S{,} \hfill \\ \end{gathered} \right.$$

and the following Main question: What are the properties of the non-classical corrector $\delta B_{AB}^{\lambda + \mu }$ (22), where $\Phi = \Phi^{\lambda A}$ is determined from the boundary-value problem (23)?

## Energy identity and inequalities for the stiffnesses

Multiplying the LHP equation in (23) by $\Phi$ ($\Phi = \Phi^{\lambda A}$**,** $L\Phi$ is defined in (18)) and then integrating twice by parts, taking into account $\Phi = \frac{\partial \Phi }{{\partial {\mathbf{n}}}} = 0$ on $S$, we obtain as a result

24 $$\int\limits_{P} {\left\{ {\left[ {\frac{1 + \nu }{E}\left( {\frac{{\partial^{2} \Phi }}{{\partial y_{3}^{2} }} - \nu\Delta \Phi } \right)} \right]\frac{{\partial^{2} \Phi }}{{\partial y_{3}^{2} }} + \left[ {\frac{1 + \nu }{E}\left( {\frac{{\partial^{2} \Phi }}{{\partial y_{2}^{2} }} - \nu\Delta \Phi } \right)} \right]\frac{{\partial^{2} \Phi }}{{\partial y_{2}^{2} }} + 2\left[ {\frac{1 + \nu }{E}\frac{{\partial^{2} \Phi }}{{\partial y_{2} \partial y_{3} }}} \right]\frac{{\partial^{2} \Phi }}{{\partial y_{2} \partial y_{3} }}} \right\}} dy_{2} dy_{3} .$$

Denote (24) $E(\Phi )$ (it has the meaning of the energy).

Multiplying the RHP equation in (23) by $\Phi$ **(**$\Phi = \Phi^{\lambda A}$**)** and then integrating twice by parts, we obtain $( - 1)^{\lambda } \int\limits_{P} {\Delta [\nu y_{A}^{\lambda } ]\Phi dy_{2} dy_{3} } = ( - 1)^{\lambda } \int\limits_{P} {\nu y_{A}^{\lambda } \Delta \Phi dy_{2} dy_{3} }$. Finally, we obtain the energy identity

25 $$E(\Phi^{\lambda A} ) = ( - 1)^{\lambda } \int\limits_{P} {\nu (y_{2} ,y_{3} )y_{A}^{\lambda } \Delta \Phi^{\lambda A} dy_{2} dy_{3} .}$$

Energy $E(\Phi ) \ge 0$ for any $\Phi$, and $E(\Phi )> 0$ if the second derivatives of $\Phi$ are nonzero. To verify this, compute the eigen values of the quadratic form in the integrand in (24). They are positive: $\lambda_{1} = 1, \, \lambda_{2} = 1 - 2\nu> 0$ since Poisson's ratio of an isotropic material $\nu \le 0.5$^4^.

From (22) and (25), we obtain $\delta D_{AB}^{\lambda + \mu } = ( - 1)^{\mu + \nu } E(\Phi^{\lambda A} )$.

### Inequalities for stiffnesses

If condition (12) is not met, the tensile stiffness $d_{{}}^{0}$ and bending stiffnesses $d_{22}^{2}$ and $d_{33}^{2}$ are greater the classical stiffness: $d_{{}}^{0}$ > $D_{{}}^{0}$, $d_{22}^{2}$ > $D_{22}^{2}$, $d_{33}^{2}$ > $D_{33}^{2}$.

This follows from the equality $d_{AB}^{\lambda + \mu }$ = $D_{AB}^{\lambda + \mu }$ + $\delta D_{AB}^{\lambda + \mu }$ and the equality of correctors $\delta D_{{}}^{0}$ ($\lambda = \mu = 0$) and $\delta D_{AA}^{2}$ ($\lambda = \mu = 1$) to the energy $E(\Phi )> 0$ (with proper $\Phi = \Phi^{\lambda A}$).

## Numerical experiments to determine the stiffnesses of a rod/beam of a coaxial structure

Let us present the results of numerical calculations confirming our theoretical conclusions and showing that the corrector $\delta D^{\lambda + \mu }$ may take very large values.

Note that the numerical solutions of the problems of elasticity theory, performed on modern FEM software, can be considered equivalent, in terms of accuracy and reliability of the results, to both analytical solutions and full-scale experiments.

### Tension

**Figure 2:**
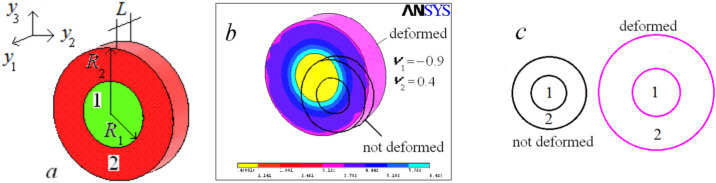
Fragment of rod of coaxial structure—(**a**), numerical solution to the problem about deformation of this fragment (tension along $Oy_{1}$-axis)—(**b**), deformation of the cross-section—(**c**).

The tensile stiffness corrector $\delta D^{0}$ is non-zero, only if the Poisson’s ratio is not a harmonic function. An example of a non-harmonic function is a piecewise-constant function. The piecewise-constant function corresponds to a rod/beam made of several homogeneous materials widely used in practice. Present our numerical solutions to the problem of tension of a cylindrical rod made of two coaxial circular cylinders, Fig. 2a. Although we reduced the original three-dimensional periodicity problem (1) to two-dimensional problem (8), solution to the problem (1) is more illustrative. In our computations $R_{1} = 1$, $R_{2} = 2$, $L = 1$, Fig. 2a. Young's moduli of both materials are taken equal to 1 (the problem is linear, and we may take Young's modules equal to 1 without loss of generality). The boundary conditions corresponding to the axial tension: displacement $u_{1} = 0$ on one side of the disk, $u_{1} = 1$ on another side. Due to the linearity of the problem, such non-physical values (calculated strains are of the order of 100%) does not lead to any problems, but makes the calculation results more visual.

Figure 2b shows the numerically computed deformed cell and the von Misess stress value. Figure 2c shows cross-sections of the rod before (left) and after (right) deformation. Note that we consider the tension of the rod and see that the diameter of the rod increases under tension. This is not very surprising since one of the components of the rod has negative Poisson's ratio. What is surprising is the giant increase in the tensile rigidity of the rod. The numerically computed tensile stiffness $d^{0}$ and ratio $\delta D^{0} {/}D^{0}$ are presented in Table 1 for various $\nu_{1}$ and $\nu_{2}$. The classical stiffness $D_{{}}^{0} = 1$.

**Table 1:** Tensile stiffnesses of rod.

$\nu_{1}$	0.3	0.1	0.4	0.3	0.45	− 0.9	− 0.99
$\nu_{2}$	0.3	0.4	0	− 0.9	− 0.95	0.4	0.49
$d_{{}}^{0}$	1	1.02	1.03	1.33	1.47	2.67	21.68
$\delta D^{0} {/}D^{0} \%$	0%	2%	3%	33%	47%	167%	2068%

The theoretical possible value of the Poisson’s ratio of isotropic materials ranges from $- 1$ to 0.5^4^. The values of Poisson’s ratio of widely used elastic isotropic materials range from 0 (cork) to 0.3 (metals) and 0.4 (plastics). The existence of materials with negative Poisson’s ratios has been confirmed both experimentally and theoretically^15,16^. Poisson's ratio of isotropic material can achieve the value $- 1$^17^. See also reviews^18–24^ and references therein.

### Bending

The problem (1) corresponding to the bending was solved for the three-dimensional cell shown in Fig. 2a. For the bending, the displacement is linearly dependent on the transverse coordinate: $u_{z} = x_{2}$ at the side $x_{1} = 2$ (here $L = 2$), and $u_{z} = 0$ on the side $x_{1} = 0$. Due to the symmetry of the beam in Fig. 2a, these boundary conditions correspond to the bending. The ratio $\delta D_{22}^{2} /D_{22}^{2}$ for various $\nu_{1}$ and $\nu_{2}$ are given in Table 2.

**Table 2:** Bending stiffnesses of beam.

$\nu_{1}$	0.4	− 0.9	− 0.95
$\nu_{2}$	0.4	0.4	0.45
$\delta D_{22}^{2} {/}D_{22}^{2} \%$	0	30%	49%

## Conclusion

The stiffnesses of rods/beams of coaxial structure coincide with the stiffnesses predicted by Bernoulli–Euler and Saint-Venant theories if and only if the condition $\Delta (\nu (y_{2} ,y_{3} )y_{A}^{\nu } ) = 0$ is satisfied.

When the condition above is not satisfied, the stiffnesses of composite rod/beam are higher than those given by the classical theories.

Differences in value of the stiffnesses given by the asymptotic theory from those given by classical theories can be significant for rods/beams formed from materials with a Poisson's ratio close to $- 1$ and close to 0.5. Although such materials are currently "exotic", their existence is theoretically justified and materials of this kind are being manufactured.

### Prospectives

Our results indicate one more potential way to produce high stiffness fibers, which have wide applications ranging from aircraft to sport equipment^25^. In the example above, the diameter of the rod decreases under the axial compression and the stiffnesses are large. Such a combination looks promising for the design of tools possessing high penetration properties. The strength of the rods/beams made of unusual materials is still an open problem. Until the strength properties are clarified, the discussed rods/beams may be recommend for design of tools penetrating into soft materials, for example, the biological matter (^26,27^ present examples of the use the materials with negative Poisson’s ratio in biomedical applications).

## Data Availability

All data is available from the corresponding author (A.A.) upon request.
